# Phase Separation Competent TIA1 Couples Glycolytic Shutdown to CD8^+^ T-Cell Activation and Shapes the Efficacy of Intravesical BCG in Bladder Cancer

**DOI:** 10.3390/biology14111576

**Published:** 2025-11-11

**Authors:** Wenwen Zhang, Kailiang Zhou, Pinru Chen, Xuanshuang Du, Min Liu

**Affiliations:** 1 Hubei Province Key Laboratory of Allergy and Immunology, Department of Immunology, Taikang Medical School (School of Basic Medical Sciences), Wuhan University, Wuhan 430071, China; 2 The Eighth Hospital of Wuhan, Wuhan 430010, China

**Keywords:** TIA1, liquid–liquid phase separation, glycolysis, CD8^+^ T cells, Bacillus Calmette–Guérin, bladder cancer

## Abstract

Metabolic immune evasion is a major factor limiting the long-term efficacy of intravesical Bacillus Calmette–Guérin (BCG) therapy in non-muscle-invasive bladder cancer (NMIBC). TIA1 is a stress granule RNA-binding protein with liquid–liquid phase separation (LLPS) capacity. Its role in tumor metabolism and immunotherapy response has been unclear. Here, we demonstrated that high TIA1 expression was independently associated with favorable survival across multiple cohorts. Full-length TIA1 formed cytoplasmic condensates, repressed LDHA/PKM2/HK2, reduced lactate, and lowered extracellular acidification. A condensate-defective ΔLCD (deletion of the low-complexity domain) mutant was inactive. TIA1 showed physical association with these glycolytic mRNAs in human cells, consistent with mRNA-linked control. Condensate-competent TIA1 promoted CD8^+^ T-cell proliferation, increased CD69 and Granzyme-B, and reduced PD-1 in co-culture. TIMER (Tumor Immune Estimation Resource) and spatial-omics supported co-localization with tumoral CD8A. BCG induced this metabolic–immune signature in cell lines, murine models, and patient explants, but the effects were abolished by TIA1 knock-down. Conversely, TIA1 over-expression alone limited tumor growth and recapitulated BCG-mediated glycolytic restraint and T-cell activation. Together, these results support an LLPS-linked, mRNA-associated regulation of tumor glycolysis. BCG-driven glycolytic suppression and CD8^+^ T cell activation track with the condensate-forming capacity of TIA1. TIA1 emerges as a prognostic biomarker and a potential therapeutic axis to improve intravesical immunotherapy in NMIBC.

## Simple Summary

Many people with early-stage bladder cancer receive bladder instillations of the tuberculosis vaccine strain, Bacillus Calmette–Guérin (BCG), yet many patients relapse. One reason is that cancer cells switch to high-rate glycolysis and release acid (lactate), which makes the body’s killer immune cells tired and less able to fight. We studied TIA1, an RNA-binding protein that forms liquid-like droplets (LLPS). We asked whether this droplet-forming ability links tumor glycolysis to CD8^+^ T-cell activity and BCG benefit. We found that people whose tumors had higher TIA1 were more likely to have better survival outcomes. In cells and mouse models, droplet competent TIA1 lowered glycolysis, reduced lactate, and increased CD8^+^ T-cell activation markers. BCG tended to enhance this pattern, and the effect depended on TIA1’s low-complexity domain. In our models, increased TIA1 expression was associated with slower tumor growth. Meanwhile, TIA1 binds glycolysis-related RNAs (LDHA, PKM2, and HK2), suggesting an mRNA-linked mechanism. Clinically, measuring TIA1 in tumor tissue may help identify patients more likely to benefit from BCG; for those with low TIA1, future strategies could consider metabolic or immune co-modulation to personalize treatment.

## 1. Introduction

Intravesical instillation of Mycobacterium Bovis Bacillus Calmette–Guérin (BCG) remains the first-line adjuvant therapy for non-muscle-invasive bladder cancer (NMIBC) and is credited with reducing recurrence and progression by 30–40% [1,2,3]. The accepted paradigm focuses on BCG-driven activation of local immunity: bladder epithelium and resident macrophages internalize bacilli, release pro-inflammatory cytokines (IL-1β, IL-6, and TNF-α), and chemokines (CXCL9/10), and recruit polymorphonuclear cells, NK cells and, ultimately, CD4^+^ and CD8^+^ T lymphocytes [4,5,6,7,8]. CD8^+^ T cells execute tumor killing via perforin/Granzyme-B, whereas Th1-polarized CD4^+^ cells amplify cytotoxicity through IFN-γ and IL-2 [9,10,11]. Despite this well-choreographed immune cascade, up to two-thirds of patients fail within five years, and accumulating evidence implicates tumor-intrinsic metabolic barriers—particularly lactate-mediated acidification—in blunting effector-cell function and promoting PD-1-driven exhaustion [12,13,14,15]. How BCG interfaces with tumor metabolism remains important but incompletely defined, and may influence clinical outcome.

Liquid–liquid phase separation (LLPS) provides cells with a rapid, reversible means to compartmentalize biochemistry without membranes [16,17,18,19]. Biomolecular condensates concentrate or exclude enzymes and RNA species, thereby controlling signal transduction, transcription, DNA repair, and metabolism [20,21,22]. Stress granules (SGs) are archetypal condensates that assemble around RNA-binding proteins such as T-cell-intracellular-antigen-1 (TIA1) under adverse conditions [23,24]. Beyond its canonical role in halting cap-dependent translation, TIA1 binds AU-rich elements, modulates alternative splicing, and has been linked to anti-viral responses, NLRP3 inflammasome suppression, and type-I-IFN signaling [25,26,27]. The low-complexity domain (LCD) of TIA1 plays a central role in facilitating SG assembly through LLPS [28]. Heterotypic interactions with stress granule co-factors Zn^2+^ and RGG-rich regions from FUS each act together with nucleic acid to induce the LLPS of TIA-1 [29]. In cancer, TIA1 dysregulation has been reported in leukemia and lung cancer [30,31,32], yet its metabolic or immunological functions remain largely unexplored, and no study has connected TIA1 to BCG responsiveness in urothelial malignancy.

In curated bladder tumor cohorts [33,34], higher TIA1 levels showed an association with favorable survival [35]. In our data, condensate-competent TIA1 aligned with a metabolic checkpoint. It was associated with lower expression of glycolytic enzymes (LDHA, PKM2, and HK2), reduced lactate secretion, and extracellular acidification, and more favorable indicators of CD8^+^-T-cell activation with lower PD-1 expression. In experimental systems, BCG exposure showed a similar pattern. The loss of TIA1 attenuated these responses in vitro, in an orthotopic MB49-Luc model, and in patient-derived NMIBC explants. Conversely, enforced expression of wild-type TIA1, but not deletion of the low-complexity domain (ΔLCD), was associated with tumor growth restraint in vivo. Collectively, our findings support an LLPS-linked, mRNA-associated repression of glycolytic transcripts with concordant CD8^+^ T cell readouts, while the exact balance between decay and translation control remains to be determined. TIA1-centered pathways may be explored to optimize intravesical immunotherapy in selected patients.

## 2. Materials and Methods

### 2.1. Animals

All animals were treated in accordance with the Institutional Animal Care and Use Committee guidelines at Wuhan University (WAEF-2022-0035; approval date: 27 February 2024). The animals were fed food and water ad libitum and housed at 22 °C with a 12 h light–dark cycle. The standard diet was provided by Xietong Biotechnology Co., Ltd. (Nanjing, China). All animal experiments were conducted in accordance with the National Institutes of Health guidelines for the care and use of laboratory animals (NIH Publications No. 8023, revised 1978). The mice were humanely killed under deep isoflurane anesthesia (4% in O_2_ for ≥3 min) followed by cervical dislocation to ensure death, in line with AVMA Guidelines for the Euthanasia of Animals (2020). Unconsciousness was confirmed by loss of corneal and pedal reflexes before the secondary method was applied.

### 2.2. Cell Culture and Reagents

Human bladder cancer lines (T24/HTB-4, UM-UC-3/CRL-1749, 5637/HTB-9, RT4/HTB-2, SW780/CRL-2169, and HT-1376/CRL-1472), HEK-293T/CRL-3216 (all ATCC), and MB49 (Sigma-Aldrich/Merck, St. Louis, MO, USA, SCC148) were authenticated by STR profiling (Genesky Biotechnologies, Shanghai, China) and confirmed to be mycoplasma-free. Cells were maintained in RPMI-1640 (or DMEM for HEK-293T) supplemented with 10% FBS (Gibco, Thermo Fisher Scientific, Waltham, MA, USA) and antibiotics at 37 °C and 5% CO_2_. Recombinant BCG (ATCC, 1 × 10^8^ CFU/vial) was reconstituted in PBS immediately before use. CFSE, ER-Tracker™ Blue/White, and C11-BODIPY 581/591 were from Invitrogen, Thermo Fisher Scientific (Waltham, MA, USA). Seahorse XF Glycolysis Stress Test kits, an l-lactate colorimetric assay (BioVision, Denver, CO, USA), and a GSH/GSSG-Glo kit (Promega, Madison, WI, USA) were used as per the manufacturers’ instructions. Antibodies are listed in Supplementary Table S1.

### 2.3. Plasmids, siRNA, and Lentiviral Transduction

Full-length human TIA1 and a ΔLCD mutant (aa 275-386 deleted) were cloned into pLVX-Puro, named as pLVX-hTIA1 and pLVX-hTIA1-ΔLCD; Full-length mouse Tia1 (Mouse Tia1 is the same length as human TIA1 (386 aa) and the C-terminal low-complexity/prion-like segment begins at the same position) and a ΔLCD mutant (aa 275-386 deleted) were cloned into pLVX-Puro [28,31], named pLVX-mTia1 and pLVX-mTia1-ΔLCD; human TIA1 (Sh-hTIA1) and mouse Tia1 shRNA (Sh-mTia1) and a non-targeting control (Sh-scramble) were inserted into pLKO.1. VSV-G-pseudotyped lentiviruses were produced in HEK-293T cells with psPAX2. Stable cell lines (TIA1-KD-T24 cells or TIA1-Over-T24 cells) were selected with puromycin (2 µg/mL) for ≥30 days.

### 2.4. RIP-qPCR

T24 cells expressing vector, TIA1-WT, or TIA1-ΔLCD were lysed in RIP buffer (150 mM KCl, 25 mM Tris-HCl pH 7.4, 5 mM EDTA, 0.5% NP-40, 1 mM DTT) with an RNase inhibitor (*n* = 3). Lysates were incubated overnight (4 °C) with anti-TIA1 or isotype IgG and protein A/G beads, washed 4× (last two at 300 mM KCl), RNA-purified, reverse-transcribed, and quantified by qPCR (primers for LDHA, PKM2, and HK2, and non-targets ACTB and HPRT1). The primers are listed in Supplementary Table S1. IgG immunoprecipitation was used as a negative control. ACTB and HPRT1 were included as non-target transcripts and showed no enrichment in anti-TIA1 IPs. Enrichment was reported as %Input and fold over IgG (ΔΔCt). Melt curves confirmed single products; no-RT controls were negative.

### 2.5. CRISPR/Cas9-Mediated Generation of TIA1-Knock-Off (KO) Cell Lines

CRISPR guide RNAs (sgRNAs) targeting human TIA1 exon 2 were designed with Benchling (https://www.benchling.com) (off-target score > 80) and cloned into lentiCRISPRv2 (Addgene #52961). The two highest-scoring guides were sgTIA1-1: 5′-GCTGGATGAGGCGCTACAGA-3′; sgTIA1-2: 5′-TCACCAACATCTCGATGAGA-3′. Target bladder cancer cells (T24) were spin-infected and selected with puromycin (2 µg mL^−1^, 7 d). Surviving pools were expanded; single-cell clones were isolated by limiting dilution where noted. Knock-down efficiency was confirmed by RT-PCR and Western blotting.

### 2.6. Proliferation, ECAR, and Lactate Assays

Cells (1 × 10^4^/well) were seeded in 96-well plates, infected or transfected as indicated and exposed to BCG (10 MOI) for 24 h (*n* = 3). Cell numbers were determined by trypan-blue exclusion. ECAR was measured on a Seahorse XF96 analyzer (Agilent Technologies, Santa Clara, CA, USA). Conditioned media were cleared by centrifugation (300 g, 5 min) and assayed for lactate. Vehicle-treated wells served as basal controls; curves were background-corrected and normalized to cell number.

### 2.7. Human CD8^+^-T-Cell Isolation and Co-Culture

CD8^+^ T cells were purified from healthy donor PBMCs by negative selection (Miltenyi Biotec, San Diego, CA, USA). After CFSE labeling, 1 × 10^5^ T cells were co-cultured with irradiated (50 Gy) bladder cancer cells (TIA1-KD-T24 cells or TIA1-Over-T24 cells) at a 1:1 ratio for 48 h (*n* = 3). The cells were harvested, stained for CD69, PD-1, and intracellular Granzyme-B (BioLegend Inc., San Diego, CA, USA) and analyzed on a BD LSRFortessa (BD Biosciences, Franklin Lakes, NJ, USA).

### 2.8. TIMER and Spatial-Omics Analyses

Correlation between TIA1 and immune cell infiltration in TCGA-BLCA was extracted with TIMER (Tumor Immune Estimation Resource) 2.0. Visium spatial-transcriptomics data (GEO GSE171351) were processed in Seurat v4; TIA1, EPCAM, and CD8A spot-level expression was visualized in ggplot2 (RStudio, Version: 2025.09.2+418).

### 2.9. Survival Analyses

Survival endpoints (OS or DFS/RFS as available) were analyzed within cohort using Cox proportional hazards models including TIA1 and available covariates (age, sex, pathological stage, and grade; adjuvant therapy when recorded). Proportional hazards assumptions were assessed (Schoenfeld residuals or graphical diagnostics). The results are reported as HR, 95% CI, and *p*. In cohorts without covariates, univariable Cox was used and labeled exploratory. TIA1 was also modeled as a continuous predictor; median splits were used for visualization only in Kaplan–Meier plots. To avoid overfitting, covariate counts were limited by events per variable. We did not pool cohorts for inference; cross-cohort graphics are descriptive.

### 2.10. Orthotopic Tumor and Intravesical BCG Model

Six- to eight-week-old female C57BL/6J mice (GemPharmatech, Nanjing, China) were instilled intravesically with 5 × 10^5^ MB49-Luc cells stably expressing Sh-Scramble or Sh-mTia1 (*n* = 6). Three days later, the mice received intravesical PBS or BCG (5 × 10^6^ CFU in 50 µL) every three days for four weeks. Tumor burden was monitored by bioluminescence imaging (IVIS Spectrum, PerkinElmer Inc., Altham, MA, USA).

### 2.11. TIA1 Gain-of-Function Models

MB49-Luc cells bearing pLVX-vector, pLVX-mTia1, or pLVX-mTia1-ΔLCD were instilled as above; the tumors were imaged on day 25.

### 2.12. Flow Cytometry of Tumor-Infiltrating Lymphocytes

The mice were humanely killed when tumor volume reached 1500 mm^3^, when body weight loss exceeded 15%, or at the predefined study end-point (day 25). Bladders (*n* = 6) were minced, digested with Collagenase IV (1 mg/mL) and DNase I (50 µg/mL) for 30 min at 37 °C, filtered through a 70 µm mesh and permeabilized, and stained with live/dead dye, CD45, CD3, CD8, CD69, PD-1, and Granzyme-B antibodies. The gating strategy is presented in Supplementary Figure S4.

### 2.13. Ex Vivo NMIBC Single-Cell Assay

Clinical fresh tumors (*n* = 6) were digested as above, resuspended at 1 × 10^6^ cells/mL in RPMI-10% FBS and incubated with PBS or BCG (1 × 10^7^ CFU/mL) for 48 h. Paired PBS and BCG conditions were run for each patient specimen; lactate was measured in supernatants, and Western blots used Tubulin as a loading control. Human specimens were obtained under approval from the Biomedical Ethics Committee of Wuhan University (Approval No. 2022007). All participants (or legal representatives) provided written informed consent using the committee-approved form. Samples were de-identified prior to research use and handled in accordance with the Declaration of Helsinki and local regulations.

### 2.14. Immunohistochemistry

Four-micron paraffin sections were deparaffinized, subjected to citrate antigen retrieval, and incubated with primary antibodies (TIA1 1:200). HRP-conjugated secondary antibodies and DAB (DAKO) were used for detection. Digital images were analyzed in Image J 2; H-scores were averaged from five random 400× fields. Archival human tissues were used under the same institutional approval (Wuhan University Biomedical Ethics Committee, Approval No. 2022007) with written informed consent. Identifiers were removed before analysis.

### 2.15. mCherry-BCG Uptake Assay and Immunofluorescence

T24 human bladder cancer cells (1 × 10^4^ cells per well) were infected with recombinant *Mycobacterium bovis* BCG constitutively expressing mCherry at a multiplicity of infection (MOI) and incubated for 4 h without additional manipulation (*n* = 3). Following infection, monolayers were washed and plasma membrane permeabilization was achieved with 0.1% Triton X-100, after which lysosomes were labeled with Lysotracker™ Green DND-26 (Thermo Fisher Scientific, Waltham, MA, USA). Nuclei were counter-stained with DAPI. Slides were mounted in ProLong™ Gold Antifade and imaged on a Zeiss LSM 880 confocal microscope using a 63×/1.4 NA objective.

### 2.16. Statistics

Unless stated, data are shown as mean ± SEM from biological replicates (cells: independent cultures; mice: animals; patients: specimens). Normality was assessed by Shapiro–Wilk test. Two-group comparisons used unpaired two-tailed Student’s *t* tests; multiple comparisons used one-way ANOVA with Tukey post hoc correction. Mouse survival curves were compared by log-rank test. Pearson correlation coefficients were calculated for TIMER data. Significance was set at *p* < 0.05. Analyses were performed in Prism 8 (GraphPad 8).

## 3. Results

### 3.1. Construction of an LLPS-Driven Prognostic Model

**Figure 1:**
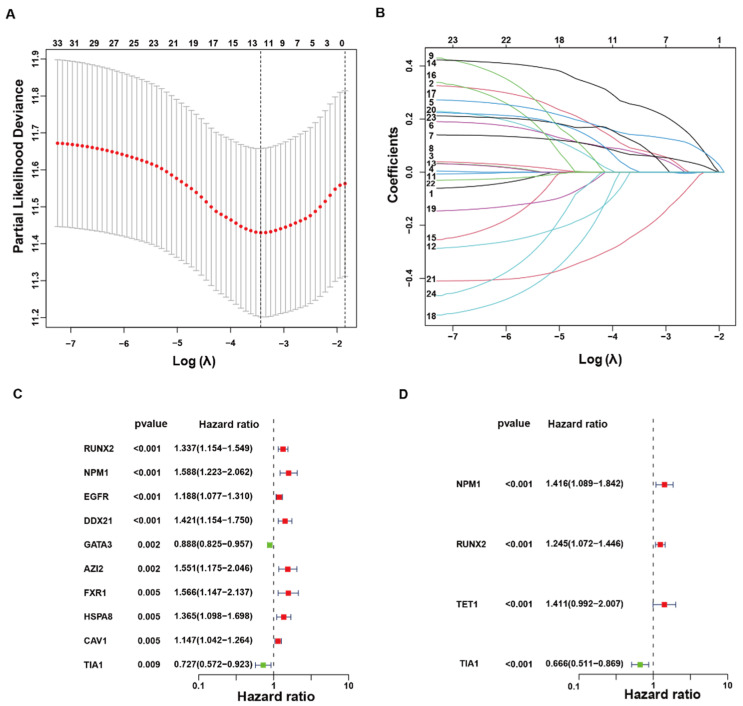
LASSO–Cox screening pinpoints a four-gene LLPS-related prognostic signature in bladder cancer. (**A**) Ten-fold cross-validation curve for the LASSO–Cox model built from 23 experimentally validated LLPS regulators in TCGA-BLCA (*n* = 408). Red points show mean partial likelihood deviance ± SEM across folds vs. log(λ). Dashed line, λ_min. Numbers above, non-zero coefficients at each λ. (**B**) Coefficient paths of the 23 candidates across log(λ); most coefficients shrink to 0 as penalization increases. (**C**) Univariable Cox forest plot of genes with non-zero coefficients at λ_min; hazard ratios (HRs) with 95% CIs are shown (log scale). Color code: red = risk (HR > 1), green = protective (HR < 1). (**D**) Multivariable Cox for genes remaining after stepwise selection; HR (95% CI) displayed. Analyses are within-cohort.

A panel of 23 LLPS regulators was extracted from the TCGA-BLCA transcriptome for survival modeling. LASSO-penalized Cox regression identified a single optimal penalty (λ_min = 0.037) at which the mean partial likelihood deviance reached its nadir and seven genes retained non-zero coefficients (Figure 1A). Progressive shrinkage of coefficients confirmed strong inter-gene collinearity, with only one predictor persisting under maximal penalization (Figure 1B). Univariate analysis ranked RUNX2 (HR = 1.34), NPM1 (HR = 1.59), EGFR, DDX21, AZ2, FXR1, HSPA8, and CAV1 as adverse factors, whereas GATA3 (HR = 0.89) and TIA1 (HR = 0.73) were protective (Figure 1C). Subsequent step-wise multivariate regression distilled the model to four independent predictors—NPM1 (HR = 1.42), RUNX2 (HR = 1.25), TET1 (HR = 1.41), and TIA1 (HR = 0.67)—thereby establishing a concise LLPS-related signature for risk stratification in bladder cancer (Figure 1D).

### 3.2. The Four-Gene Signature Robustly Stratifies Patient Survival

**Figure 2:**
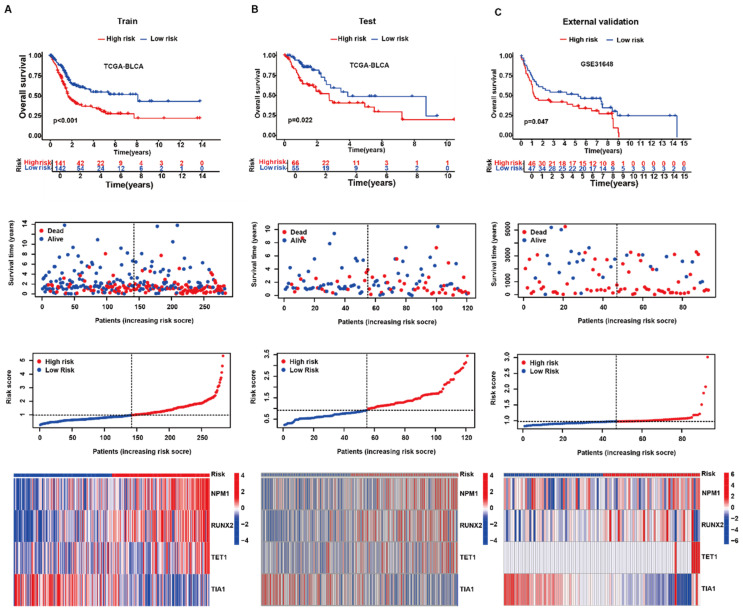
Validation of the four-gene LLPS-related risk signature in internal and external bladder cancer cohorts. (**A**) Training set (TCGA-BLCA). Top: Kaplan–Meier curves for overall survival (OS) in high- vs. low-risk groups defined by the median risk score; risk table shown. Middle: survival-status scatter by increasing risk score (red, deceased; blue, alive); gray dashed line, median cut-off. Bottom: ranked risk-score distribution and matched heat map of z-scored expression for NPM1, RUNX2, TET1, and TIA1. (**B**) Internal test set (TCGA-BLCA). Same layout and cut-off as in (**A**); OS separation reproduced. (**C**) External validation (GSE31684, *n* = 93). Same layout; the signature stratifies OS in an independent cohort. KM curves and log-rank *p* are for display only; inference is from Cox models reporting HR (95% CI). Heat map values are per-gene z-scores. Two-sided tests; analyses within cohort.

Applying the risk formula derived from NPM1, RUNX2, TET1, and TIA1 to the TCGA-BLCA training subset (70% random split) separated patients into distinct prognostic strata: the high-risk group had a markedly shorter median OS (29.4 months) than the low-risk group (not reached; log-rank *p* < 0.001; Figure 2A, upper). A clear monotonic increase in death events was observed along the risk-score gradient, whereas low-risk patients were predominantly alive at last follow-up (Figure 2A, middle). Consistently, the expression heat map revealed up-regulation of the three adverse genes (NPM1, RUNX2, and TET1) and down-regulation of the protective gene (TIA1) in high-risk tumors (Figure 2A, lower). The model maintained discriminatory power in the remaining 30% of TCGA samples (test set); high-risk patients exhibited a significant survival disadvantage (hazard ratio [HR] = 1.89, 95% CI 1.09–3.29; *p* = 0.022; Figure 2B). External validation using the GSE31684 cohort reproduced these findings, with high-risk cases showing poorer OS (HR = 1.78, 95% CI 1.01–3.17; *p* = 0.047; Figure 2C). The coherent ordering of gene expression patterns and the progressive rise in risk scores across all three datasets underscore the robustness and transferability of the LLPS-driven prognostic signature.

### 3.3. LLPS-Related Risk Score Is an Independent Predictor and Improves Clinical Prognostication

**Figure 3:**
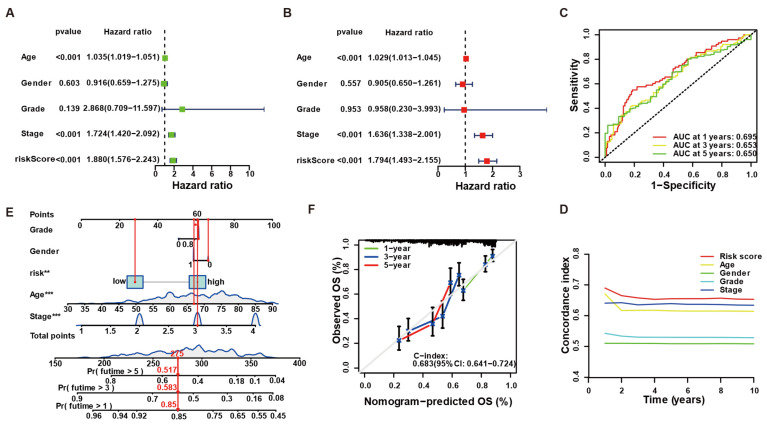
Prognostic analyses of the LLPS-based risk score in bladder cancer (within-cohort). (**A**) Univariable Cox forest plot for overall survival (OS) in TCGA-BLCA (*n* = 408). Squares show hazard ratios (HRs); horizontal bars show 95% confidence intervals (CIs) for age, sex, grade, stage, and the LLPS-derived risk score. (**B**) Multivariable Cox including the same covariates; the risk score remains significant after adjustment (HR with 95% CI shown). (**C**) Time-dependent ROC curves for 1-, 3-, and 5-year OS; AUCs = 0.695, 0.653, 0.650, respectively. (**D**) Time-varying C-index comparing the risk score with individual clinical factors across follow-up. (**E**) Nomogram combining risk score, age, sex, and stage to estimate 1-, 3-, and 5-year OS. (**F**) Calibration of the nomogram at 1/3/5 years (bootstrap-corrected); the 45° gray line denotes perfect agreement. Overall C-index = 0.684 (95% CI 0.641–0.724). KM plots (when shown) are for display only; inference by Cox with HR (95% CI). Significance: ** *p* < 0.01, *** *p* < 0.001.

In within-cohort univariate analysis, the LLPS-based risk score was strongly associated with poorer overall survival (HR = 1.88, 95% CI 1.58–2.24, *p* < 0.001; Figure 3A). After adjusting for age, gender, grade, and stage, the risk score retained prognostic significance (HR = 1.79, 95% CI 1.49–2.16, *p* < 0.001; Figure 3B), underscoring its independence from conventional variables. Time-dependent ROC analysis yielded AUCs of 0.695, 0.653, and 0.650 at 1, 3, and 5 years, respectively (Figure 3C), indicating stable predictive power over time. Consistently, dynamic C-index curves demonstrated that the risk score outperformed individual clinicopathological factors throughout the entire follow-up period (Figure 3D). To illustrate bedside use, we built a within-cohort nomogram incorporating the risk score, age, gender, and stage (Figure 3E). Bootstrap-corrected calibration curves showed excellent agreement between predicted and observed survival at all time points, and the nomogram achieved a C-index of 0.684 (95% CI 0.641–0.724; Figure 3F). All analyses were performed within the cohort shown and adjusted for available covariates. Risk-group splits are used for visualization only; inference relies on Cox models. We did not pool cohorts for statistical inference.

To explore the biological processes underlying risk stratification, we compared transcriptomes between high- and low-risk patients. GO over-representation analysis revealed that high-risk tumors preferentially expressed genes related to cytokine activity, immune-receptor binding, and collagen organization, whereas low-risk tumors were enriched for extracellular matrix (ECM) organization and chemotaxis (Supplementary Figure S1A,B). Consistent with these findings, GSEA showed significant enrichment of pro-inflammatory pathways—cytokine–cytokine receptor interaction and focal adhesion—in the high-risk cohort (normalized enrichment score [NES] > 2.0, FDR < 0.01; Supplementary Figure S1C). In contrast, the low-risk group exhibited enrichment of metabolic and epithelial integrity pathways, including oxidative phosphorylation and ECM–receptor interaction (NES < −2.0, FDR < 0.01; Supplementary Figure S1D). These results provide context, suggesting that adverse risk aligns with inflammatory/ECM-remodeling programs, whereas favorable risk aligns with metabolic and epithelial integrity programs.

### 3.4. Risk Group-Specific Transcriptional Programs Implicate Cytokine Signaling and Metabolic Homeostasis

**Figure 4:**
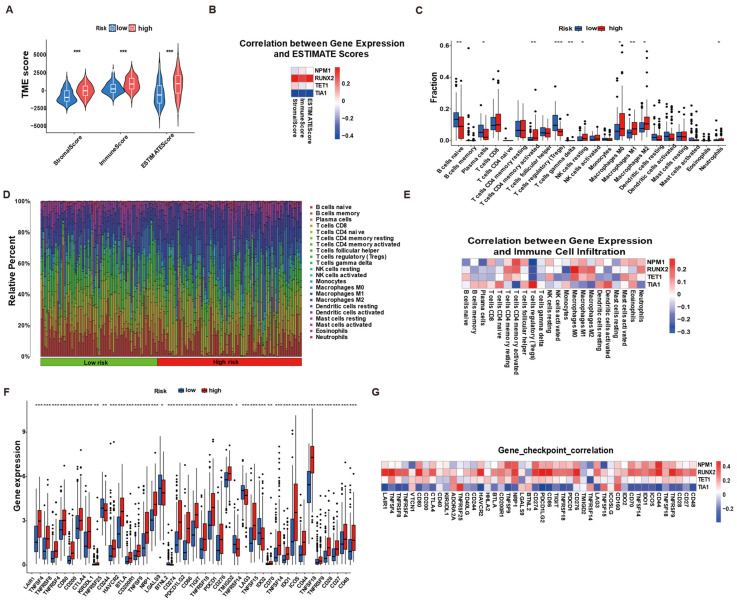
Global functional profiling distinguishes high- and low-risk tumors defined by the LLPS-based gene signature. (**A**) Violin plots of ESTIMATE Stromal, Immune, and Total scores comparing low- vs. high-risk groups. Center line = median; box = IQR; whiskers = 1.5 × IQR. Two-tailed Wilcoxon test. (**B**) Heat map of Pearson correlations between the four signature genes (NPM1, RUNX2, TET1, and TIA1; expression in log_2_(TPM + 1)) and the three ESTIMATE scores; color denotes r. (**C**) Box-and-whisker plots of ssGSEA infiltration scores for 28 immune populations in low- vs. high-risk tumors; Wilcoxon test with Benjamini–Hochberg (BH) correction. (**D**) Stacked bar plot of the relative abundance (%) of 22 CIBERSORT leukocyte subsets per patient, ordered from low- (green bar) to high-risk (red bar). (**E**) Heat map of Pearson correlations (r) between the four genes and the 22 CIBERSORT subsets (blue = negative; red = positive). (**F**) Box plots of 29 immune-checkpoint transcripts (log_2_(TPM + 1); axis truncated as shown). (**G**) Gene–checkpoint correlation matrix (Pearson r) for the same panel. Statistics: two-tailed tests; multiple comparisons adjusted by BH where applicable. Significance: * *p* < 0.05, ** *p* < 0.01, *** *p* < 0.001; ns, not significant.

To uncover biological mechanisms underlying the prognostic separation, we compared transcriptomes of high- versus low-risk tumors. Low-risk tumors, which are enriched for the protective gene TIA1, displayed significantly higher Stromal Score, Immune Score, and global ESTIMATE Score than high-risk counterparts (Figure 4A, ** *p* < 0.001). Concordantly, TIA1 expression correlated positively with all three scores, whereas the three adverse genes (NPM1, RUNX2, and TET1) correlated negatively (Figure 4B). Immune-cell profiling reinforced these differences. CIBERSORT and ssGSEA revealed greater proportions of activated CD8^+^ T cells, M1-like macrophages, and NK cells in the low-risk group, whereas high-risk tumors were relatively enriched for M2 macrophages, resting mast cells, and immune-suppressive neutrophils (Figure 4C,D). Gene–cell correlations confirmed that TIA1 aligned most strongly with effector populations (CD8^+^ T cells, activated memory CD4^+^ T cells), while NPM1, RUNX2, and TET1 associated with immunosuppressive subsets (Figure 4E). These microenvironmental differences translated into divergent checkpoint profiles. Low-risk tumors expressed higher levels of co-stimulatory molecules (e.g., CD27 and ICOS) and lower levels of inhibitory ligands such as PD-L1 and LAG3, whereas the opposite pattern characterized the high-risk cohort (Figure 4F). At the gene level, TIA1 showed positive correlations with multiple co-stimulatory checkpoints and negative correlations with key inhibitory receptors, in stark contrast to the adverse genes (Figure 4G). Together these data indicate that the poor-prognosis, high-risk signature is embedded in a “cold” cytokine-rich but immunosuppressed microenvironment, whereas the favorable low-risk group features a metabolically restrained, CD8^+^-T-cell-inflamed niche—providing an immunological explanation for the survival advantage conferred by the TIA1-dominated LLPS signature.

### 3.5. TIA1 Suppresses Lactate Production and Glycolytic Enzyme Expression via Its Low-Complexity Domain

**Figure 5:**
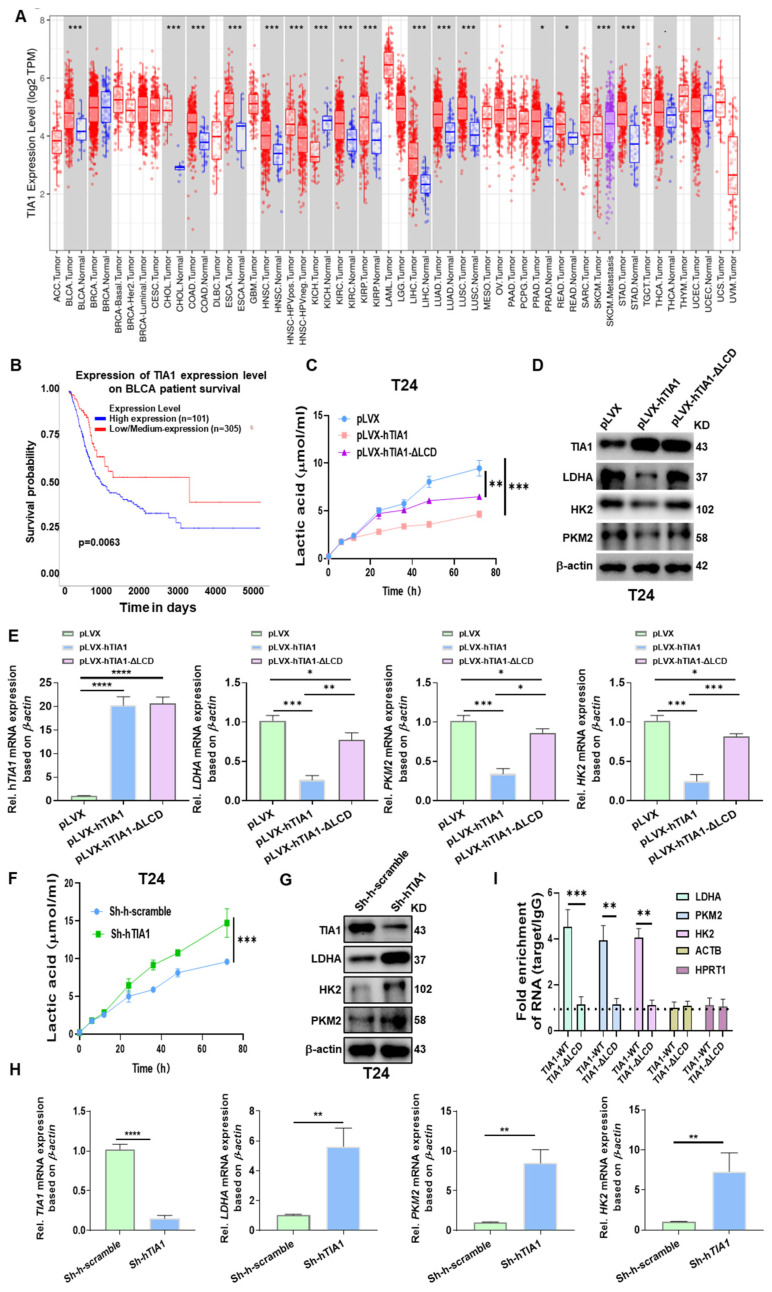
TIA1 constrains glycolysis in bladder cancer cells and requires condensate competence. (**A**) Pan-cancer TIA1 expression in paired tumor (red: TCGA primary tumors; purple: TCGA metastatic samples, SKCM—metastasis) vs. adjacent normal (blue) tissues from TCGA (33 types). Boxes = median with IQR; dots = individual samples; gray shading marks tumor types without matched normal. Two-tailed Wilcoxon test. (**B**) Kaplan–Meier overall survival in TCGA-BLCA stratified by median TIA1 (high, red; low, blue); log-rank *p* shown. (**C**) Time course of extracellular lactate (μmol·mL^−1^) in T24 cells expressing empty vector (pLVX), wild-type TIA1 (pLVX-TIA1), or a low-complexity domain deletion (ΔLCD; LLPS-impaired control). Data are mean ± SEM, *n* = 3 independent experiments; two-way ANOVA with Šidák correction. (**D**) Immunoblots of TIA1, LDHA, HK2, and PKM2 in the same lines; β-actin, loading control. Blots are representative of ≥3 independent experiments. (**E**) qRT-PCR for TIA1, LDHA, PKM2, and HK2 (log_2_ fold vs. control) in pLVX, TIA1-WT, and TIA1-ΔLCD cells. Mean ± SEM, *n* = 4; one-way ANOVA (Tukey). (**F**) Lactate release over 80 h (μmol·mL^−1^) in Sh-scramble vs. Sh-TIA1 T24 cells. Mean ± SEM, *n* = 3; two-way ANOVA. (**G**) Immunoblots for TIA1 and glycolytic enzymes in sh-scramble and sh-TIA1 cells (representative of ≥3 experiments). (**H**) Densitometry of LDHA, PKM2, and HK2 following TIA1 knock-down (normalized to β-actin). Mean ± SEM, *n* = 4; unpaired two-tailed *t*-test. (**I**) RIP–qPCR showing TIA1 association with glycolytic mRNAs in T24 cells. Bars show fold enrichment over IgG for LDHA, PKM2, and HK2 in TIA1-WT vs. TIA1-ΔLCD; dashed line = IgG baseline (=1). Mean ± SEM, *n* = 3; one-way ANOVA (Tukey). Significance for all panels: * *p* < 0.05, ** *p* < 0.01, *** *p* < 0.001, **** *p* < 0.0001 (two-tailed).

Pan-cancer analysis of TCGA revealed that TIA1 is broadly expressed, with significantly higher transcript levels in bladder tumors than in matched normal urothelium (Figure 5A). Consistently, elevated TIA1 predicted prolonged overall survival in TCGA-BLCA (log-rank *p* = 0.0003; Figure 5B). Immunohistochemistry in 40 paired specimens showed a marked reduction in the TIA1 protein in tumor epithelium compared with adjacent normal tissue (median H-score 38 vs. 125; **** *p* < 0.0001; Supplementary Figure S2A,B), underscoring its clinical relevance. Functional experiments confirmed a direct metabolic role for TIA1.

Since the LCD of TIA1 plays a central role in facilitating SGs assembly through LLPS, we used the ΔLCD variant (LCD deletion) as a condensation-deficient control. Enforced expression of full-length TIA1 in T24 cells curtailed extracellular lactate accumulation and suppressed LDHA, HK2, and PKM2 at both the mRNA and protein level, whereas the domain-deletion control-ΔLCD variant produced only modest effects (Figure 5C–E). Conversely, shRNA knock-down doubled lactate release and up-regulated the same glycolytic enzymes (Figure 5F–H), demonstrating that endogenous TIA1 restrains aerobic glycolysis.

Loss-of-function and rescue assays strengthened these findings (Supplementary Figure S2B–D). CRISPR knockout in T24 cells recapitulated the glycolytic phenotype; lactate secretion rose sharply and glycolytic-gene transcripts increased. Re-introduction of wild-type TIA1—but not the ΔLCD mutant—reversed both lactate production and LDHA/PKM2/HK2 expression to baseline (Supplementary Figure S2B–D).

Anti-TIA1 RIP–qPCR in T24 cells demonstrated significant enrichment of glycolytic mRNAs in TIA1-WT immunoprecipitates compared with isotype IgG (mean fold over IgG ± SEM: LDHA ~5×, PKM2 ~3.5–4×, HK2 ~3–3.5×; all *p* < 0.01; *n* = 3). In contrast, ΔLCD showed near-baseline enrichment (≤1.5×). Non-target transcripts (ACTB and HPRT1) exhibited no enrichment (~1.0–1.3×). These data provide direct evidence that condensate-competent TIA1 associates with key glycolytic mRNAs (Figure 5I).

Collectively, these results demonstrate that TIA1 suppresses the glycolytic program in bladder cancer cells, and that this repression is largely dependent on its low-complexity domain and condensate-forming capacity. While our data support a model of LLPS-linked post-transcriptional repression, distinguishing ARE-mediated decay from translational attenuation will require additional assays.

### 3.6. BCG Suppresses Tumor Glycolysis in a TIA1-Dependent Manner Across Human and Murine Bladder Cancer Models

**Figure 6:**
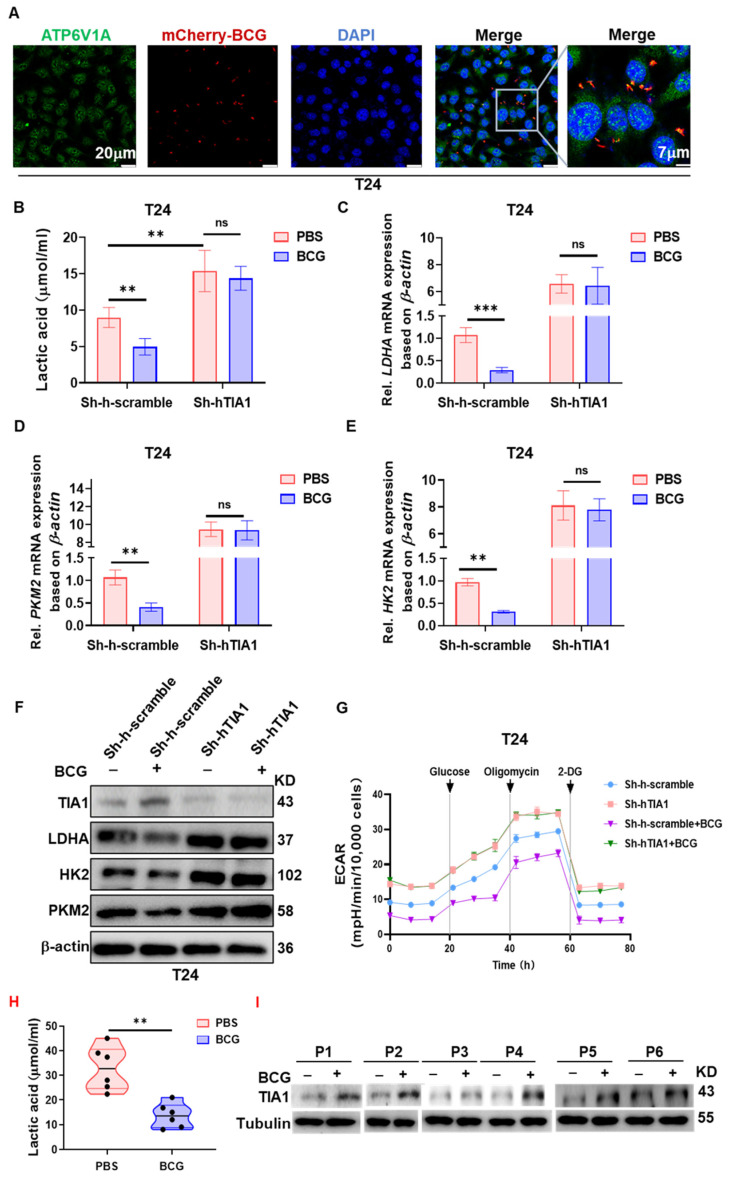
TIA1 is required for BCG-mediated repression of glycolysis in bladder cancer cells. (**A**) Confocal images of T24 cells incubated for 4 h with mCherry-labeled BCG (red). Endolysosomal ATP6V1A (green); nuclei, DAPI (blue). Right panels show higher-magnification insets. Scale bars: overview, 20 µm; inset, 7 µm. (**B**) Extracellular lactate (μmol·mL^−1^) in Sh-scramble vs. Sh-TIA1 cells treated with PBS or BCG (1 × 10^7^ CFU·mL^−1^, 24 h). Mean ± SEM, *n* = 3 independent experiments; two-way ANOVA with Šidák correction. (**C**–**E**) qRT-PCR of LDHA (**C**), PKM2 (**D**), and HK2 (**E**) under the same conditions as (**B**). Fold vs. PBS control after β-actin normalization. Mean ± SEM, *n* = 4; two-way ANOVA (Šidák). (**F**) Immunoblots of TIA1, LDHA, HK2, and PKM2 in Sh-scramble vs. Sh-TIA1 cells ± BCG; β-actin, loading control. Blots representative of ≥3 experiments. (**G**) Seahorse ECAR profiles after BCG. Sequential injections: glucose, oligomycin, 2-deoxy-glucose (vertical dotted lines). Curves show mean ± SEM of 5 technical replicates; representative of 3 independent experiments. (**H**,**I**) Ex vivo NMIBC single-cell assay (*n* = 6 patients). Dissociated fresh tumors cultured with PBS or BCG (1 × 10^7^ CFU·mL^−1^, 48 h). (**H**) Supernatant lactate (each dot = one patient); paired two-tailed test as indicated. (**I**) Representative immunoblots of TIA1 (Tubulin, loading control) for matched PBS/BCG in P1–P6. Statistics: two-tailed; significance: ** *p* < 0.01, *** *p* < 0.001; ns, not significant.

Because intravesical BCG is instilled into an intact bladder lumen, the bacilli make first contact not with professional immune cells but with the tumor epithelium itself. Recent work shows that live BCG can enter urothelial carcinoma cells within minutes, triggering transcriptional remodeling before a systemic immune response is mounted [36]. Given our finding that condensate-competent TIA1 is a potent brake on glycolysis, we asked whether BCG could exploit the same axis to impose a metabolic ‘checkpoint’ on bladder cancer cells, thereby creating a micro-environment conducive to subsequent CD8^+^-T-cell attack. To test this hypothesis, we exposed human and murine bladder cancer lines to mCherry-labeled BCG in the absence of immune effectors and monitored lactate production, glycolytic enzyme expression, and the real-time extracellular acidification rate (ECAR). Confocal imaging confirmed that mCherry-labeled BCG is efficiently taken up into ATP6V1A-positive vesicles of T24 cells (Figure 6A). Concordantly, BCG down-regulated LDHA, PKM2 and HK2 mRNA, and protein by roughly half in Sh-scramble cells, whereas knock-down cells showed no change (Figure 6C–F). Seahorse analysis revealed a parallel functional shift: BCG lowered basal ECAR and glycolytic capacity by ~40% in Sh-scramble cells, yet curves for Sh-hTIA1 cells were super-imposable with or without BCG (Figure 6G).

The breadth and TIA1-dependence of this phenomenon were confirmed in Supplementary Figure S3. In six additional human bladder cancer lines (5637, HT-1376, RT4, SW780, TCCSUP, and UM-UC3), BCG consistently decreased lactate release, although the magnitude varied between cell types (Supplementary Figure S3A). Extending the analysis to the murine MB49 model, BCG reduced lactate secretion and suppressed LDHA/PKM2/HK2 only in sh-scramble controls, whereas TIA1 knock-down completely negated these changes (Supplementary Figure S3B–E).

To provide human context, freshly dissociated NMIBC tumors (n = 6) cultured ex vivo with BCG for 48 h showed lower lactate in supernatants and higher TIA1 protein in cell lysates (Figure 6H,I).

Overall, these results are consistent with a BCG-associated attenuation of aerobic glycolysis in bladder cancer cells that depends, at least in part, on TIA1. They also align cellular and animal findings with ex vivo human evidence, strengthening the case for a tumor cell-intrinsic metabolic component to BCG action.

### 3.7. TIA1 Is Associated with BCG-Linked Glycolytic Restraint and CD8^+^-T-Cell Activation in Bladder Cancer

**Figure 7:**
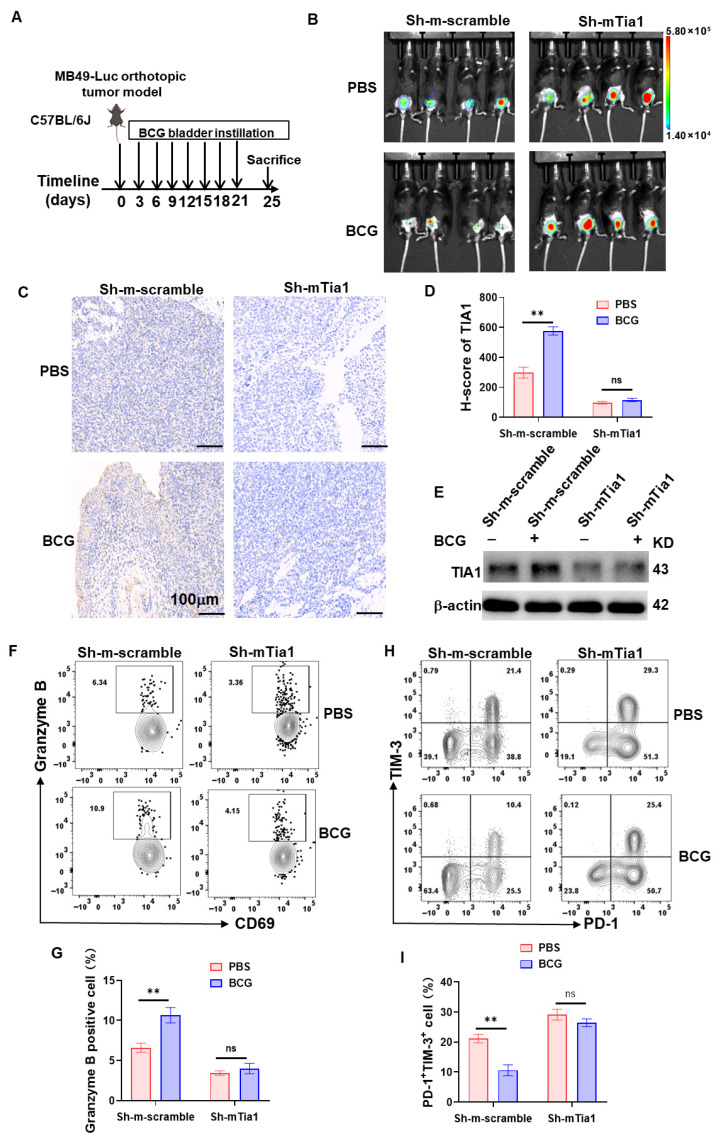
TIA1 knock-down negates the anti-tumor and immuno-metabolic effects of intravesical BCG in an orthotopic MB49-Luc bladder-cancer model. (**A**) Scheme. C57BL/6J mice received orthotopic MB49-Luc cells expressing Sh-scramble or Sh-TIA1. From day 3, intravesical PBS or BCG weekly ×4; necropsy day 25. (**B**) Representative bioluminescence imaging (BLI) on day 25. (**C**) H&E of bladder sections; scale bar, 100 µm. (**D**) Tumor burden: total photon flux (photons·s^−1^) and TIA1 IHC H-score. Mean ± SEM, n = 6 per group; two-way ANOVA (Šidák). (**E**) Tumor lysate immunoblot of TIA1; β-actin, loading control (representative of ≥2 experiments). (**F**) Flow-cytometry dot plots of Granzyme-B vs. CD8 in TILs. (**G**) %Granzyme-B^+^ of CD8^+^ TILs. Mean ± SEM, *n* = 3; two-way ANOVA (Šidák). (**H**) Flow plots of PD-1 vs. TIM-3 on CD8^+^ TILs. (**I**) %PD-1^+^TIM-3^+^ “exhausted” CD8^+^ T cells. Mean ± SEM; statistics as in (**G**). Statistics: two-tailed; significance: ** *p* < 0.01; ns, not significant.

To determine whether the TIA1-dependent, anti-glycolytic effect we observed in vitro also operates in a physiologically intact, immune-competent setting, we turned to an orthotopic MB49-Luc bladder tumor model in C57BL/6J mice. Over-expression of wild-type, condensate-forming TIA1 (pLVX-mTia1) in orthotopic MB49-Luc tumors markedly reduced bioluminescence and bladder-mass size 21 days after implantation, whereas vector control and the low-complexity domain mutant (TIA1-ΔLCD) grew unchecked (Supplementary Figure S4A). In the converse loss-of-function setting, weekly intravesical BCG suppressed tumor radiance, histological burden, and glycolytic enzyme expression only in Sh-scramble animals (Figure 7A–E). When TIA1 was silenced, BCG lost its efficacy: Sh-mTia1 tumors retained intense photon flux (Figure 7B), persistent tumor architecture (Figure 7C), high LDHA/HK2/PKM2 levels (Figure 7E), and unaltered lactate output. Immunophenotyping mirrored the metabolic data. BCG treatment elevated the proportion of activated CD69^+^Granzyme-B^+^ and reduced exhausted PD-1^+^ TIM-1^+^CD8^+^ tumor-infiltrating lymphocytes in control mice (Figure 7F–I; gating scheme in Supplementary Figure S4). These immunological benefits were completely absent in TIA1-deficient tumors, underscoring the requirement for endogenous TIA1. Additionally, single-cell suspensions from freshly resected NMIBC specimens secreted ~40% less lactate after 48 h of BCG stimulation and simultaneously up-regulated intracellular TIA1 protein (Supplementary Figure S4B).

Collectively, the gain-of-function, loss-of-function, and patient-derived data all suggested that TIA1 condensates may constitute the molecular switch that couples BCG exposure to tumor cell glycolytic repression and revitalization of CD8^+^ T cell anti-tumor immunity.

### 3.8. Correlation and Spatial Convergence of TIA1 with Cytotoxic T-Cell Immunity

**Figure 8:**
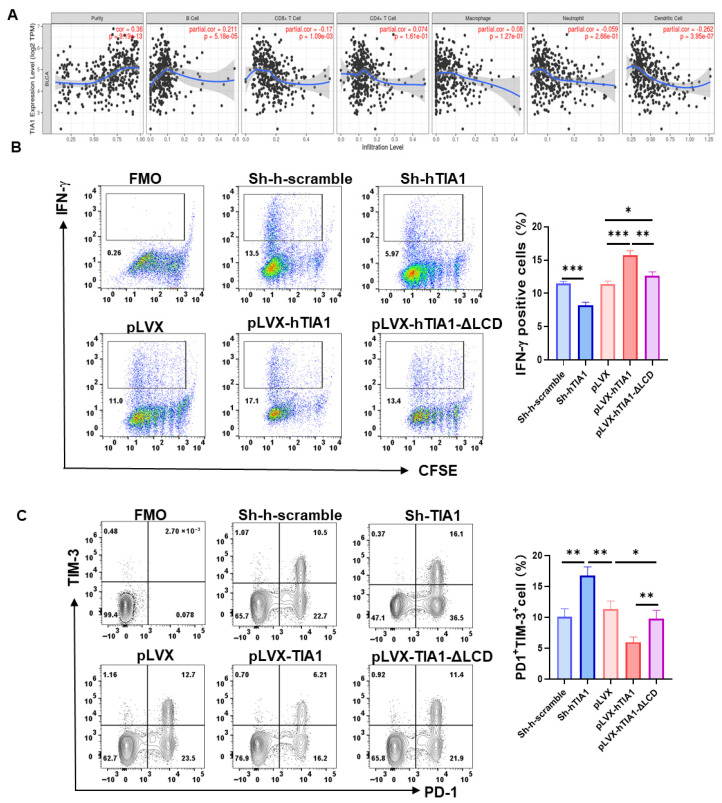
TIA1 correlates with CD8^+^-T-cell infiltration in bulk tumors and enhances CD8^+^-T-cell activation in co-culture. (**A**) TIMER 2.0 scatter plots showing the relationship between TIA1 mRNA (log_2_[TPM + 1]) and estimated infiltration of seven immune populations in TCGA-BLCA (Purity, B cells, CD8^+^ T cells, CD4^+^ T cells, macrophages, neutrophils, dendritic cells). Dots, individual tumors; blue band, 95% confidence of LOESS fit. Pearson r and *p* reported by TIMER. Note: analyses are correlative and descriptive; no causal inference is drawn. (**B**) Representative flow-cytometry density plots of CFSE-labeled human peripheral-blood CD8^+^ T cells after 48 h co-culture with bladder cancer cells (empty vector, TIA1-WT, or ΔLCD). Numbers indicate % proliferating CD8^+^ T cells. (**C**) Frequencies of CD69^+^ and PD-1^+^ within the CD8^+^ compartment. Mean ± SEM, *n* = 3 donors; one-way ANOVA with Tukey post hoc test. Statistics: two-tailed; significance: * *p* < 0.05, ** *p* < 0.01, *** *p* < 0.001. Note: ΔLCD is an LLPS-impaired proxy control.

We then quantified this in TCGA-BLCA. Bulk deconvolution showed that higher TIA1 correlated with all effector leukocyte populations, strongest for CD8^+^ T cells (Figure 8A; Pearson r = 0.31, *p* = 2.6 × 10^−12^). TIA1 also showed an inverse correlation with tumor purity, consistent with greater overall immune infiltration in TIA1-high tumors. To test whether TIA1 can directly modulate CD8^+^-cell function, we co-cultured CFSE-labeled human CD8^+^ T cells with T24 bladder cancer cells engineered for TIA1 gain- or loss-of-function. Wild-type TIA1 over-expression (pLVX-hTIA1) nearly doubled the proportion of IFN-γ-producing CD8^+^ cells (17 ± 1%) compared with vector (11 ± 1%), whereas the condensate-defective ΔLCD mutant was only modestly stimulatory (13 ± 1%). TIA1 knock-down markedly blunted IFN-γ induction (6 ± 1%; Figure 8B). Exhaustion profiling gave the reciprocal pattern: PD-1^+^ TIM-3^+^ double-positive cells comprised 18 ± 2% of the CD8^+^ compartment after co-culture with vector cells but fell to 9 ± 1% with TIA1-WT. ΔLCD cells failed to suppress exhaustion (15 ± 2%), and Sh-hTIA1 cultures showed the highest exhaustion frequency (24 ± 2%; Figure 8C). All differences were significant (*p* < 0.05, *n* = 3). Spatial transcriptomics analysis of four adjacent sections from a human bladder carcinoma further underscored the link between TIA1 and cytotoxic immunity (Supplementary Figure S5B). Spots with high TIA1 expression co-localized with epithelial EPCAM^+^ areas that were densely populated by CD8A transcripts, producing a recurring overlay pattern in all sections examined. Together with the bulk TIMER plots (Figure 8A), these data converge on the conclusion that TIA1-rich tumor islands recruit and activate CD8^+^ T cells, and that condensate-competent TIA1 within cancer cells is required to sustain this immunogenic dialog.

Collectively, our functional assays demonstrate that LLPS-impaired, domain-deletion TIA1 orchestrates a dual program of tumor glycolytic suppression and CD8^+^-T-cell activation, positioning TIA1 as a link that may integrate metabolic control with anti-tumor immunity in bladder cancer.

## 4. Discussion

Classical BCG emphasize innate recruitment and Th1-polarized immunity that enable CD8^+^ activity [37]. The existing literature indicates that glycolysis and lactate can suppress these responses in NMIBC [38]. Our results showed that condensate-competent TIA1 aligns with lower glycolytic output and stronger CD8^+^ markers in vitro, in an orthotopic model, and in ex vivo limited NMIBC samples. In curated cohorts, higher TIA1 is associated with better survival. Our TIMER and spatial analyses are presented as supportive correlations only, we interpret these links as associative within our systems, not as proof of a single causal pathway.

A LLPS-restricted screen pinpointed TIA1 as the only condensate regulator whose high expression independently predicted favorable survival across five cohorts. Condensate-competent TIA1, but not ΔLCD, repressed glycolytic enzymes (LDHA, PKM2, and HK2), lowered lactate secretion, and fostered CD8^+^-T-cell proliferation, CD69 up-regulation, and PD-1 down-modulation in co-culture. Meanwhile, BCG instillation reproduced this metabolic–immune signature in vitro, in an orthotopic MB49-Luc model and, ex vivo, in patient NMIBC specimens; all effects were lost upon TIA1 knock-down. These results align with a TIA1-linked pattern rather than as exclusivity. Conversely, enforced expression of wild-type TIA1 alone curtailed orthotopic tumor growth, establishing sufficiency. Collectively, the data suggest that TIA1 condensation may locate at the nexus of tumor metabolism and adaptive immunity.

To directly connect condensate competence with glycolytic repression, we performed anti-TIA1 RIP–qPCR in human T24 cells, which showed significant enrichment of LDHA, PKM2, and HK2 mRNAs in TIA1-WT immunoprecipitates versus IgG, whereas TIA1-ΔLCD exhibited near-baseline enrichment; non-targets (ACTB/HPRT1) were not enriched (Figure 5I). These findings are consistent with an mRNA-associated mechanism. However, the present dataset does not distinguish whether repression primarily reflects ARE-mediated decay or reduced translational engagement within condensates. Definitive resolution will require 3′UTR/ARE mutagenesis, RIP/CLIP-seq, and polysome profiling or SUnSET readouts in future work.

TIA1’s canonical role is to nucleate stress granules and stall translation under stress [27,28]. The coherence between tumor-intrinsic metabolic phenotypes (lactate/ECAR), decreased glycolytic enzyme expression, and TIA1–mRNA association suggests that TIA1-dependent condensates are a hypothesis-generating axis rather than a validated therapeutic target at present. A pragmatic discovery path would involve high-content imaging of condensate metrics under glycolytic stress, orthogonal 3′UTR reporter, and lactate/ECAR counters, together with toxicity/SG-over-activation counter-screen to yield chemical probes.

Several condensate-forming proteins link stress responses to metabolism. G3BP1–driven stress granules modulate translation during acute stress. HuR and TTP are ARE-binding RBPs with opposite effects [39,40]: HuR stabilizes transcripts, whereas TTP promotes decay [41,42]. hnRNPA1 regulates splicing and translation and can phase separate [43,44]. Metabolic enzymes can also cluster or condense, which may tune pathway flux; glycolytic enzymes show such clustering in stress [45]. These reports provide context for our TIA1 findings [29,46]. In our study, TIA1 binds glycolytic mRNAs, and its effects track with condensate competence under BCG conditions. This positions TIA1 as a candidate RNA-binding protein linking metabolic RNA handling to immune-relevant outputs in bladder cancer, consistent with the broader condensate literature. We did not benchmark TIA1 against these regulators. Whether TIA1 acts alone or cooperates with other scaffolds remains unknown and will require side-by-side perturbations in future work.

This study has several limitations that should be addressed in future research. We analyzed survival within cohort and did not pool datasets for inference. Model covariates were limited by events-per-variable to reduce overfitting; datasets with modest event counts are reported as exploratory and interpreted cautiously. A sensitivity analysis treating TIA1 as a continuous predictor yielded the same direction of effect as dichotomized displays. We use MB49 and multiple human cell lines, yet intravesical BCG is administered clinically almost exclusively in NMIBC; confirmation in additional in vivo NMIBC models (e.g., orthotopic xenografts of human lines) is warranted. Second, the upstream signals that modulate TIA1 condensation in bladder cancer remain undefined; cytokine- or hypoxia-driven phosphorylation could alter phase behavior and merits investigation. Third, the patient cohort used for ex vivo assays was small, and prospective trials correlating TIA1 status with BCG response are needed to validate clinical utility. Third, we used the ΔLCD variant (deletion of the low-complexity domain, aa 275–386) as an LLPS-impaired control. Because large domain deletions may alter protein–protein/RNA interactions beyond phase behavior, ΔLCD serves as an operational tool rather than a definitive test of LLPS causality. Future specificity tests will employ point mutants that perturb multivalency without removing the domain (e.g., aromatic-to-alanine substitutions in the LCD; phosphomimetic/abolishing variants), and optoDroplet gain-/loss-of-condensation assays, alongside 3′UTR ARE mutagenesis and polysome/SUnSET readouts. Forth, we did not detect large early differences in innate markers (such as upstream macrophage/neutrophil cytokine kinetics), and that definitive resolution will require a focused study. Finally, resolving whether TIA1 primarily drives ARE-dependent decay versus translational attenuation will require 3′UTR/ARE mutagenesis and polysome-level assays in future studies. These steps are planned for follow-up work and will refine the mechanistic model.

## 5. Conclusions

Our findings recast TIA1—from a classical stress granule component to a gatekeeper of immunometabolism that aligns with BCG-associated tumor control in our models. By integrating phase separation biology with metabolic and immune checkpoints, the work supports a condensate-linked regulatory axis and motivates hypothesis-driven strategies: stabilizing or mimicking TIA1-dependent glycolytic restraint should be explored cautiously to potentially enhance intravesical BCG and other immunotherapies in bladder cancer pending further validation.

## 6. Patients

Primary non-muscle-invasive bladder cancer (NMIBC) specimens were obtained from six patients undergoing transurethral resection at the Department of Urology, Renmin Hospital (Wuhan, China) in 2024–2025. None had received intravesical therapy before surgery. All participants provided informed consent, and the study was approved by the Ethics Committee of the Life Sciences Medical Ethics Committee of Wuhan University (Approval No. 20220007; Approval Date: 7 March 2022). Tumors were either fixed in 10% neutral-buffered formalin and paraffin-embedded for immunohistochemistry (IHC) or mechanically dissociated, enzymatically digested to single-cell suspensions, and cultured ex vivo with PBS or BCG (1 × 10^7^ CFU/mL) for 48 h for lactate and Western blot analyses.

## Data Availability

The original contributions presented in this study are included in the article and its Supplementary Materials. No new datasets were generated during the current study. Further inquiries can be directed to the corresponding author.

## Supplementary Materials

The following supporting information can be downloaded at: https://www.mdpi.com/article/10.3390/biology14111576/s1, Figure S1: Functional enrichment separating high- vs low-risk groups; Figure S2: TIA1 KO-and-rescue validate condensate-dependent glycolytic repression; Figure S3: TIA1-dependent glycolytic suppression by BCG; Figure S4: In vivo TIA1 overexpression and CD8⁺ gating strategy; Figure S5: Spatial co-localization of TIA1 with EPCAM and CD8A. Table S1. The primer sequences for PCR. All data generated or analyzed during this study are included in this published article and its Supplementary Information Files. Additional datasets are available from the corresponding author upon reasonable request. Public datasets used in this study include TCGA-BLCA (https://www.cancerimagingarchive.net/collection/tcga-blca, accessed on 10 July 2024) and GEO (GSE171351).

## Abbreviations

The following abbreviations are used in this manuscript:

BCG	Bacillus Calmette–Guérin
NMIBC	Non-muscle-invasive bladder cancer
LCD/ΔLCD	Low-complexity domain/deletion of the low-complexity domain
LLPS	Liquid–liquid phase separation
SGs	Stress granules
TIA1	T-cell-intracellular-antigen-1
TIMER	Tumor Immune Estimation Resource
IHC	Immunohistochemistry
ECAR	Extracellular Acidification Rate
