# Supplementary material for: Phase Separation Competent TIA1 Couples Glycolytic Shutdown to CD8+ T-Cell Activation and Shapes the Efficacy of Intravesical BCG in Bladder Cancer
Source: Biology (Basel). 2025 Nov 11;14(11):1576. doi: 10.3390/biology14111576 (PMC12649904; doi:10.3390/biology14111576)
Supplement: Supplementary file 1 [file biology-14-01576-s001.zip › biology-3928602-supplementary.pdf]

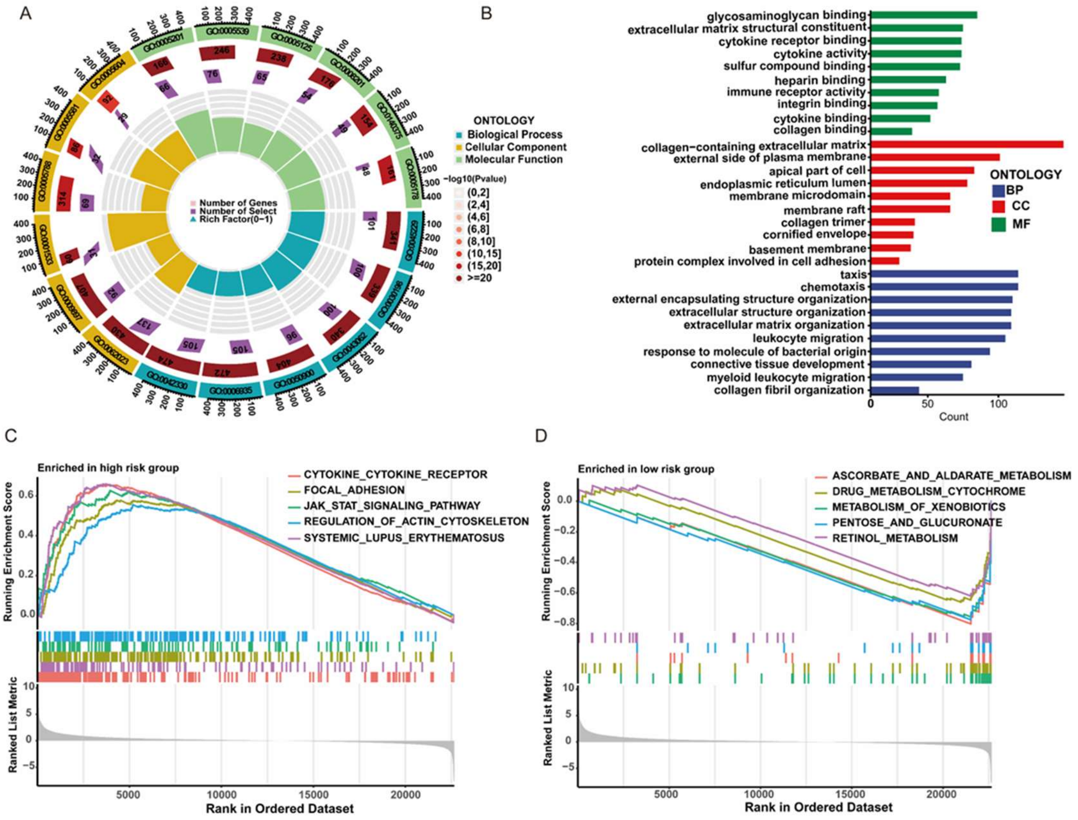

Figure S1. Functional programs distinguishing high- vs low-risk groups defined by the LLPS-related signature. (A) Circular plot summarizing differentially expressed genes (DEGs) between high- and low-risk BLCA tumors (TCGA-BLCA), grouped by functional categories. (B) GO over-representation analysis (biological process, cellular component, molecular function) showing top enriched terms; bars denote –log10(adjusted *p*). (C) GSEA enrichment curves for pathways up in the high-risk group (e.g., cytokine–cytokine receptor interaction, focal adhesion); (D) pathways up in the low-risk group (e.g., oxidative phosphorylation, ECM–receptor interaction). Statistics: two-sided tests; multiple comparisons adjusted by Benjamini–Hochberg where applicable; GSEA reports NES and FDR.

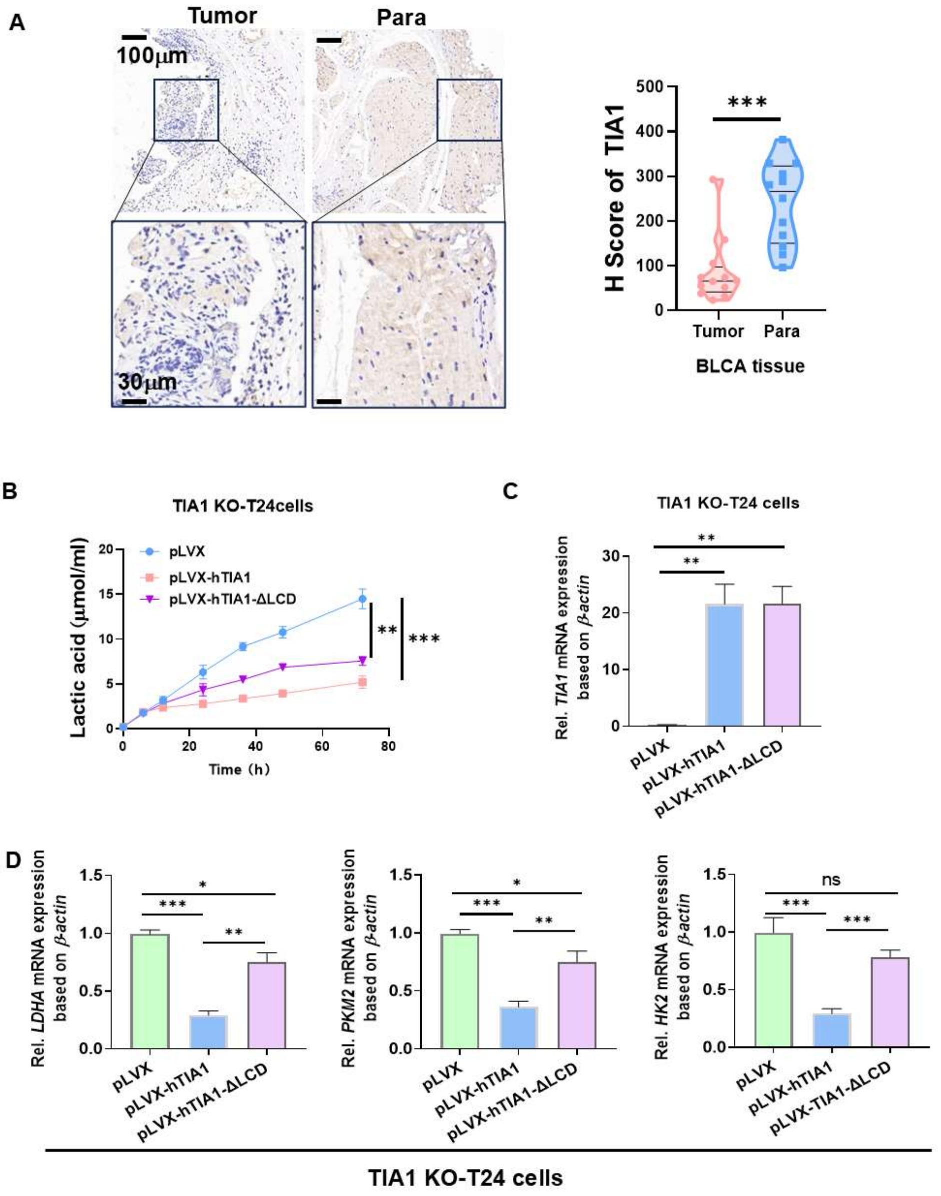

Figure S2. Loss- and rescue-of-function confirm TIA1-dependent repression of glycolysis in T24 cells. (A) Representative IHC images of TIA1 in paired human BLCA tissues (Tumor vs Para; scale bars as indicated) with H-score comparison. (B) Time-course of extracellular lactate after reintroduction of vector (pLVX), wild-type TIA1 (pLVX-hTIA1), or the condensation-defective ΔLCD variant into TIA1-KO T24 cells. (C–D) qRT-PCR of TIA1, LDHA, PKM2 and HK2 (relative to β-actin) under the same conditions. Data are mean ± SEM from independent cultures; one-/two-way ANOVA with appropriate post-hoc tests; significance: *p<0.05, **p<0.01, ***p<0.001; ns, not significant.

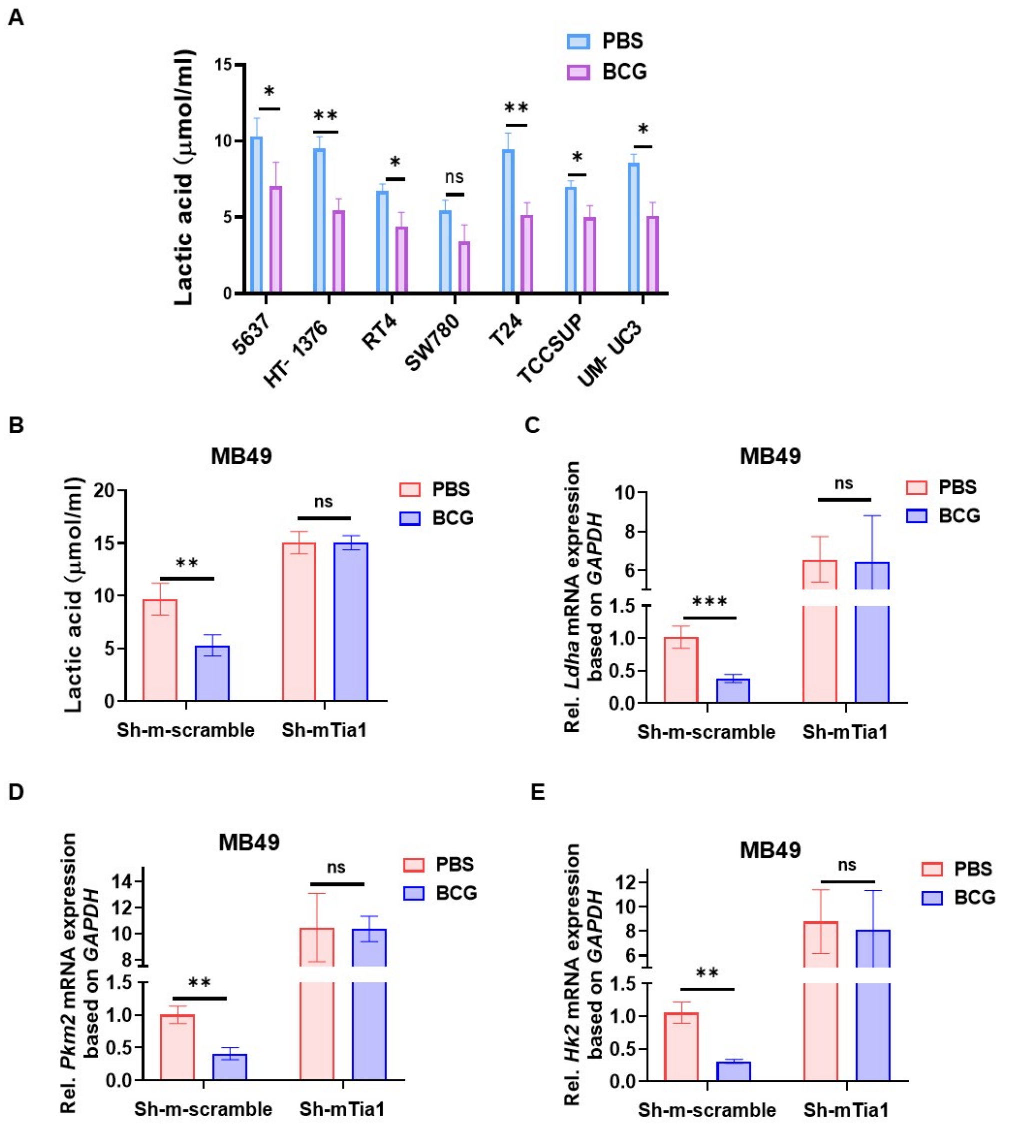

Figure S3. BCG lowers tumor-cell glycolysis in a TIA1-dependent manner across human lines and the murine MB49 model. (A) Lactate release (μmol·mL⁻¹) in six additional human BLCA cell lines (5637, HT-1376, RT4, SW780, TCCSUP, UM-UC3) after PBS vs BCG; bars = mean ± SEM; two-tailed tests with BH correction across lines. (B–E) Murine MB49 cells with sh-scramble or sh-mTia1: extracellular lactate and mRNA of Ldha, Pkm2, Hk2 following PBS vs BCG. BCG reduces lactate and glycolytic-gene expression in controls but not when Tia1 is silenced, indicating TIA1 dependency. Data are mean ± SEM from independent cultures; one-/two-way ANOVA with appropriate post-hoc tests; significance: *p<0.05, **p<0.01, ***p<0.001; ns, not significant.

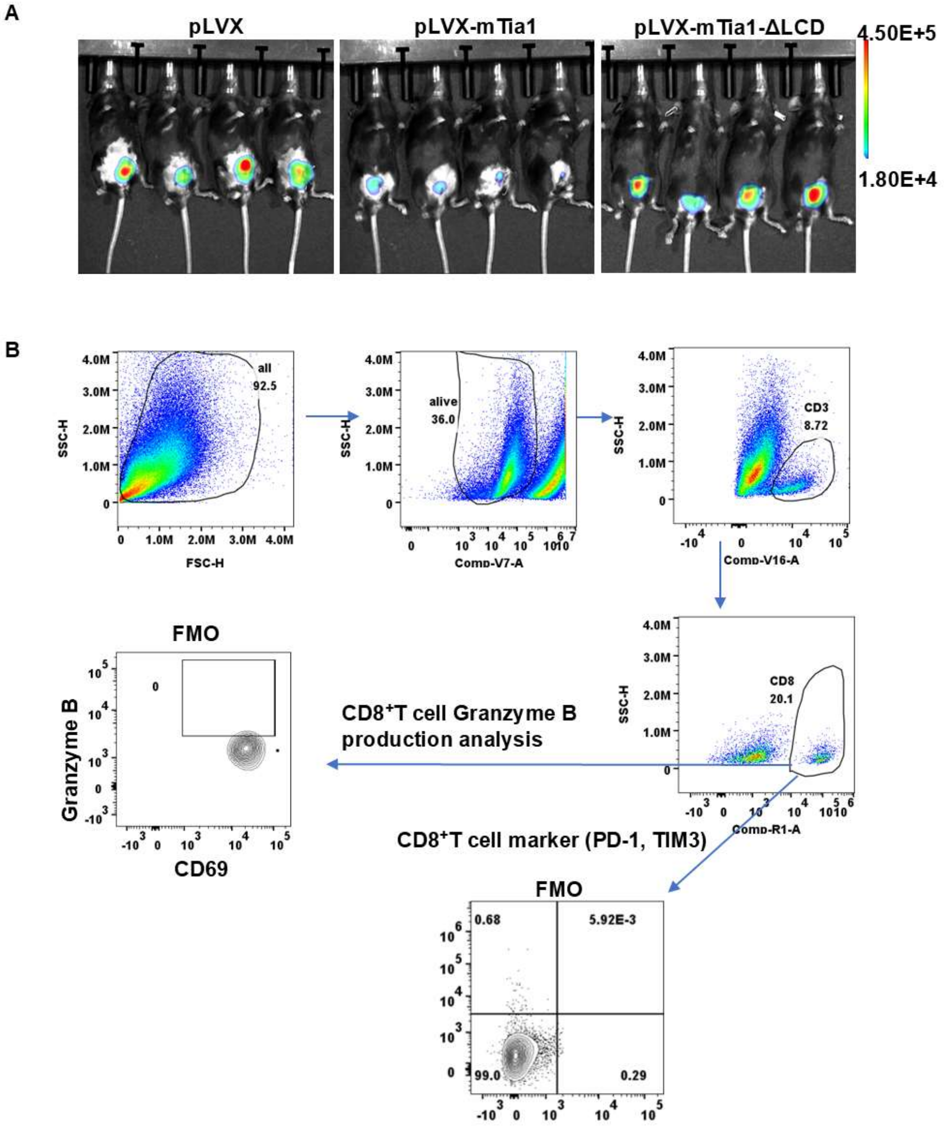

Figure S4. In vivo gain-of-function and flow-cytometry gating strategy for CD8+ T-cell readouts. (A) Orthotopic MB49-Luc tumors overexpressing vector, mTia1-WT or mTia1-Δ LCD: representative bioluminescence imaging at endpoint (color bar indicates photon flux). Overexpression of condensate-competent TIA1 reduces tumor burden relative to vector and ΔLCD controls. (B) Flow-cytometry gating scheme used throughout: FSC/SSC → singlets → live CD45+CD3+CD8+ lymphocytes; example gates for Granzyme-B vs CD69 and for PD-1 vs TIM-3 are shown; FMO controls indicated. These gates underlie the quantifications reported in the main-text figures.

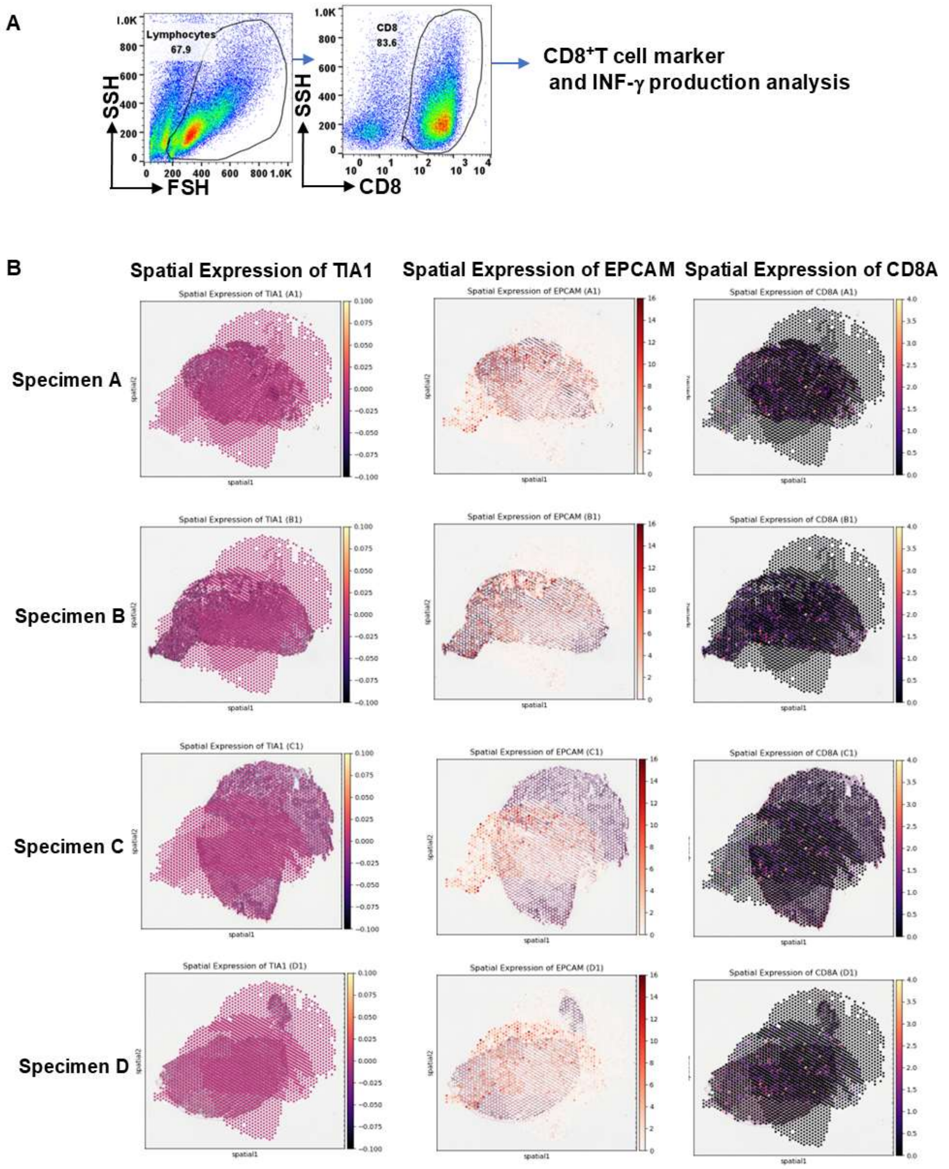

Figure S5. Convergent human evidence: CD8⁺ markers and spatial co-localization of TIA1 with epithelial/immune niches. (A) Representative flow-cytometry plots illustrating CD8⁺ T-cell identification and IFN-γ production analysis (gating sequence and FMO controls as indicated). (B) Spatial-transcriptomics maps from four human bladder-cancer specimens (A–D): spot-level expression of TIA1, EPCAM (epithelial regions) and CD8A (cytotoxic T-cell marker). Areas of high TIA1 repeatedly co-localize with EPCAM⁺ tumor islets enriched for CD8A, supporting the association between TIA1 and cytotoxic infiltration observed in bulk analyses. Color scales are dataset-normalized per panel.

Supplementary Table S1. The primer sequences for PCR.

Gene	Forward primer (5′→3′)	Reverse primer (5′→3′)
**TIA1**	GCCTAATGGTTGGCAAGTTCCTG	CCATTTTGCCCTTGAGGCGGTT
**LDHA**	ATGGCAACTCTAAAGGATCAGC	CCAACCCCAACAACTGTAATCT
**PKM2**	ATAACGCCTACATGGAAAAGTGT	TAAGCCCATCATCCACGTAGA
**HK2**	GAGCCACCACTCACCCTACT	CCAGGCATTCGGCAATGTG
**Tia1**	CAGTGGCTTGGTGGAAGACAA	TGGGTCTGACGAACAAATGAG
**Ldha**	TGTCTCCAGCAAAGACTACTGT	GACTGTACTTGACAATGTTGGGA
**Pkm**	CCAAGAGGTGAGTGCTTCCC	CTGTTGTTCAGACTCTCTCCCT
**Hk2**	ATGATCGCCTGCTTATTCACG	CGCCTAGAAATCTCCAGAAGGG
